# The Efficacy of Daily Local Antibiotic Lavage via an Epidural Suction–Irrigation Drainage Technique in Spondylodiscitis and Isolated Spinal Epidural Empyema: A 20-Year Experience of a Single Spine Center

**DOI:** 10.3390/jcm12155078

**Published:** 2023-08-02

**Authors:** Mido Max Hijazi, Timo Siepmann, Ibrahim El-Battrawy, Percy Schröttner, Dino Podlesek, Kay Engellandt, Gabriele Schackert, Tareq A. Juratli, Ilker Y. Eyüpoglu, Andreas Filis

**Affiliations:** 1 Technische Universität Dresden, Faculty of Medicine, and University Hospital Carl Gustav Carus, Department of Neurosurgery, Division of Spine Surgery, Fetscherstrasse 74, 01307 Dresden, Germany; dino.podlesek@ukdd.de (D.P.); gabriele.schackert@ukdd.de (G.S.); tareq.juratli@ukdd.de (T.A.J.); ilker.eyuepoglu@ukdd.de (I.Y.E.); afilis@neuromaster.gr (A.F.); 2 Technische Universität Dresden, Faculty of Medicine, and University Hospital Carl Gustav Carus, Department of Neurology, Fetscherstrasse 74, 01307 Dresden, Germany; timo.siepmann@ukdd.de; 3 Bergmannsheil University Hospitals Bergmannsheil, Ruhr University Bochum, Department of Cardiology, Bürkle de la Camp-Platz 1, 44789 Bochum, Germany; ibrahim.elbattrawy2006@gmail.com; 4 Technische Universität Dresden, Faculty of Medicine, and University Hospital Carl Gustav Carus, Institute for Microbiology and Virology, Fetscherstrasse 74, 01307 Dresden, Germany; percy.schroettner@ukdd.de; 5 Technische Universität Dresden, Faculty of Medicine, and University Hospital Carl Gustav Carus, Institute of Diagnostic and Interventional Neuroradiology, Fetscherstrasse 74, 01307 Dresden, Germany; kay.engellandt@ukdd.de

**Keywords:** spondylodiscitis, isolated spinal epidural empyema, epidural suction–irrigation drainage, surgical site infection, complications, relapse

## Abstract

Background: Various treatment modalities are available for local antibiotic therapy in spondylodiscitis (SD) and isolated spinal epidural empyema (ISEE), but there is no evidence-based recommendation. Postoperative epidural suction–irrigation drainage (ESID) is thought to reduce bacterial load, which may prevent the development of relapse, wound healing, hematogenous spread, and systemic complications. We evaluated the efficacy of postoperative ESID over 20 years on disease progression and outcome in SD and ISEE. Methods: Detailed demographic, clinical, imaging, laboratory, and microbiological characteristics were examined in our cohorts of 208 SD and ISEE patients treated with and without ESID at a university spine center in Germany between 2002 and 2022. Between-group comparisons were performed to identify meaningful differences for the procedure. Results: We included data from 208 patients (142 SD, 68.3% vs. 66 ISEE, 31.7%) of whom 146 were ESID patients (87 SD, 59.6% vs. 59 ISEE, 40.4%) and 62 were NON-ESID patients (55 SD, 88.7% vs. 7 ISEE, 11.3%). ESID patients with SD showed more frequent SSI (ESID: 22, 25.3% vs. NON-ESID: 3, 5.5%, *p* = 0.003), reoperations due to empyema persistence or instability (ESID: 37, 42.5% vs. NON-ESID: 12, 21.8%, *p* = 0.012), and a higher relapse rate (ESID: 21, 37.5% vs. NON-ESID: 6, 16.7%, *p* = 0.037) than NON-ESID patients with SD. The success rate in NON-ESID patients with SD was higher than in ESID patients with SD (ESID: 26, 29.9% vs. NON-ESID: 36, 65.6%, *p* < 0.001). Multivariate binary logistic regression analysis showed that ESID therapy (*p* < 0.001; OR: 0.201; 95% CI: 0.089–0.451) was a significant independent risk factor for treatment failure in patients with SD. Conclusions: Our retrospective cohort study with more than 20 years of experience in ESID technique shows a negative effect in patients with SD in terms of surgical site infections and relapse rate.

## 1. Introduction

Successful treatment of primary spinal infections (PSI), including spondylodiscitis (SD) and isolated spinal epidural empyema (ISEE), is a clinical challenge and can be addressed with a variety of surgical techniques [1]. Once the pathogen is identified, the focus is on systematic therapy, whereby the use of local antibiotic irrigation during and after surgery is an important adjunctive treatment [2]. The primary purpose of local antibiotic irrigation is to reduce the bacterial load, which may prevent the development of recurrence, wound healing, hematogenous spread, and systemic complications by shortening the duration of antibiotic therapy [2].

Several techniques for local antibiotic irrigation have been reported, but none of them has yet gained acceptance. Some authors reported on a small number of patients with continuous or onetime epidural irrigation and drainage as an effective method to reduce the residual infection and the risk of nosocomial complication [3,4,5]. Other authors proposed CT-guided percutaneous needle drainage with irrigation showing its efficacy in ISEE [6]. The endoscopic application in this setting has increased recently, with many authors reporting posterolateral endoscopic debridement followed by percutaneous drainage for irrigation [7,8].

One disadvantage of local antibiotic therapy using collagen carriers or antibiotic irrigation is increased seroma formation [9,10]. Löhr et al. reported an increased risk of epidural fluid stasis following the use of postoperative suction–irrigation drainage with no beneficial effects on outcome [11]. It is not known whether local antibiotic irrigation after surgical debridement promotes fusion [2].

Postoperative epidural suction–irrigation drainage (ESID) has been reported to have potential advantages and disadvantages; however, previous studies did not examine the short- and long-term outcome of ESID on SD and ISEE. The purpose of this study was to determine the efficacy of postoperative ESID on disease course and outcome on SD and ISEE, and to identify the benefits and drawbacks of this technique.

## 2. Materials and Methods

### 2.1. Study Design and Patient Data

#### 2.1.1. Study Design

Retrospective data from 208 patients with SD and ISEE managed with ESID or NON-ESID were analyzed. Consecutive patients with PSI who underwent surgery at our neurosurgical university spine center in Dresden, Germany, between 2002 and 2022 were included. Patients with early or late postoperative secondary spinal infections, patients with intradural infections (subdural abscess or spinal cord abscess) on admission, or patients with only conservative treatment were excluded from this study.

#### 2.1.2. Institutional Review Board and Electronic Patient Data Software

The study was approved by the local ethics committee of the Medical Faculty of the TU Dresden and University Hospital Carl Gustav, Dresden (Ref: BO-EK-17012022). Data on patients were identified via the ORBIS system (ORBIS, Dedalus, Bonn, Germany) and imaging files via the IMPAX system (IMPAX, Impax Asset Management Group plc, London, UK).

#### 2.1.3. Patient Data

Electronic medical records were pseudonymized and first divided into two groups—ISEE or SD—according to the type of infection. In addition, we examined the SD and ISEE groups to determine whether patients were treated with ESID or not (NON-ESID). Thereby, we formed 4 groups.

Baseline data were analyzed, along with age, gender, type of antibiotic therapy strategy (empirical or targeted), presence of paravertebral psoas abscess or pleural abscess, localization of infection in the spine, type of bacterial group, primary infectious source, time to surgery, time to pathogen detection, incidental dural tears, intraoperative local antibiotic irrigation, and type of surgery such as only abscess evacuation, one- or two-stage surgery.

In a next step, risk factors such as immunosuppression, diabetes mellitus, obesity, malignancy, liver cirrhosis, dialysis, stent or vascular prosthesis, artificial valve replacement, osteoporosis, rheumatoid arthritis or increased rheumatoid factors, gout or increased uric acid, chronic venous insufficiency, peripheral artery disease, and atrial fibrillation were analyzed.

Subsequently, disease-related complications such as sepsis, septic embolism, meningism, endocarditis with vegetations, surgical site infections (SSI), reoperations due to SSI, reoperations due to persistent empyema or increased spinal instability, relapse rate, mortality, length of hospital and intensive care unit (ICU) stay, duration of intravenous antibiotic administration, and total duration of antibiotic administration were analyzed.

We determined the success rate in the PSI and SD groups with and without ESID, whereas treatment failure was present in cases of persistent empyema, instability, SSI with or without reoperation, relapse rate, or death. Last, we determined the independent risk factors in multivariate binary logistic regression analysis for treatment failure. Sex, age over 65 years, methicillin-sensitive *Staphylococcus aureus* (MSSA), time to pathogen detection, empirical antibiotic treatment (EAT), time to surgery, diabetes mellitus, hepatic cirrhosis, malignancy, paravertebral psoas abscess, pleural abscess, incidental dural tears, and ESID were evaluated in multivariate binary logistic regression analysis.

### 2.2. Clinical Management and Microbiological Assessment

#### 2.2.1. Assessment of Clinical, Microbiological, and Radiological Diagnostics

We determined the diagnosis of SD or ISEE based on the medical history; clinical examination; fever; laboratory values such as leukocyte count, C-reactive protein (CrP), and Procalcitonin; typical radiological changes on magnetic resonance imaging (MRI) and computed tomography (CT); and pathogen detection in blood cultures, intraoperative specimens, or CT-guided biopsies of paravertebral psoas abscess. A transoesophageal echocardiogram (TEE) was obtained in patients with suspected infective endocarditis according to the modified DUKE criteria.

Depending on the clinical condition, at least two blood cultures were taken before antibiotic therapy was started for microbiological diagnosis, although some patients were initially treated with antibiotics due to their severe clinical condition. Tissue samples collected during open surgery and samples obtained by CT-guided biopsies were used for microbiological analysis.

In our multidisciplinary spine board or neurosurgical–neuroradiology conference, the diagnosis of ISEE or SD was performed depending on clinical, laboratory, and radiological findings. From 2002 to 2015, cases were discussed in our neurosurgical–neuroradiology conference and treated in collaboration with infectiologists when possible. Since 2015, we have established a multidisciplinary spine board that includes neuroradiologists, neurosurgeons, trauma surgeons, orthopedic surgeons, and infectiologists when appropriate in order to determine the best treatment strategy for patients.

#### 2.2.2. Antibiotic and Surgical Management

##### Surgical Treatment

First-line treatment was usually conservative with intravenous antibiotics, although surgical treatment was indicated in cases of source control, epidural abscess, neurologic deficits, or spinal instability. The type of surgical intervention was determined in the multidisciplinary spine board or neurosurgical–neuroradiology conference. All of our patients in this study required surgery and were treated with various surgical procedures.

Patients with ISEE underwent abscess evacuation with ESID or anterior cervical discectomy and fusion (ACDF) for abscesses ventral to the cervical spinal cord with ESID. Patients with SD were treated with either abscess evacuation alone or one- or two-stage instrumentation due to spinal instability, deformity, and pain-related immobility. Therefore, surgical decision-making depends on clinical experience and various defined radiological features. To assess bony integrity, preoperative CT scans were obtained from all patients who received spinal instrumentation.

##### Choice between ESID and NON-ESID Techniques in ISEE and SD

Of the 66 ISEE patients, only 7 did not receive ESID. In reviewing the medical records, no clear reason was identified explaining why the surgeons opted for NON-ESID. In 2 cases, the ESID was removed immediately postoperatively when the patient was repositioned. Incidental dural tears or reactive tissue without evidence of pus as the cause have not been reported in the remaining cases. The placement of an ESID in SD patients was based on surgeon experience and intraoperative findings, although patients treated with single-stage surgical abscess evacuation and simultaneous fixation were treated up to 81% without an ESID.

##### Epidural Suction–Irrigation Drainage

During abscess evacuation in patients with SD and ISEE, we placed one or two ESIDs in the spinal canal, when possible, with one ESID inserted cranially and one caudally. Beginning on the first postoperative day, the ESIDs were irrigated twice daily with gentamycin (5 mg in 5 mL), and samples were collected daily from the irrigation drainages for microbiological examination before irrigation. In case the identified pathogen was resistant to gentamycin, ESIDs were irrigated with vancomycin whenever possible. During irrigation, the suction drainage was closed and reopened after half an hour. If there were three bacteria-free microbiological results from the ESIDs on three consecutive days, the ESIDs were removed, and in most cases, the drainages were removed between postoperative days 8 and 14.

##### Antibiotic Treatment

Either targeted antibiotic treatment (TAT) or EAT was administered to all patients, depending on the clinical condition at admission and according to the recommendations from the local infectious disease department. However, it is important to mention that the majority of patients with EAT presented to us from secondary hospitals and required immediate surgery, therefore the number of EAT patients was high. The EAT was switched to TAT after detection of the causative pathogens.

In case of SD, intravenous antibiotic therapy was administered for about 4–6 weeks and then switched to oral antibiotics for total duration of 10 and 12 weeks. On the other hand, ISEE patients received intravenous antibiotic treatment for about 2 weeks and then switched to oral administration for a total duration of 4 to 6 weeks. Follow-up clinical and radiological examinations were conducted in all patients who complied with our recommendation at 3, 6, and 12 months after hospital discharge, if possible.

### 2.3. Illustrative Cases

We selected two different cases to describe our strategy for managing patients with ISEE and SD using ESID.

#### 2.3.1. First Case with Isolated Spinal Epidural Empyema

**Figure 1:**
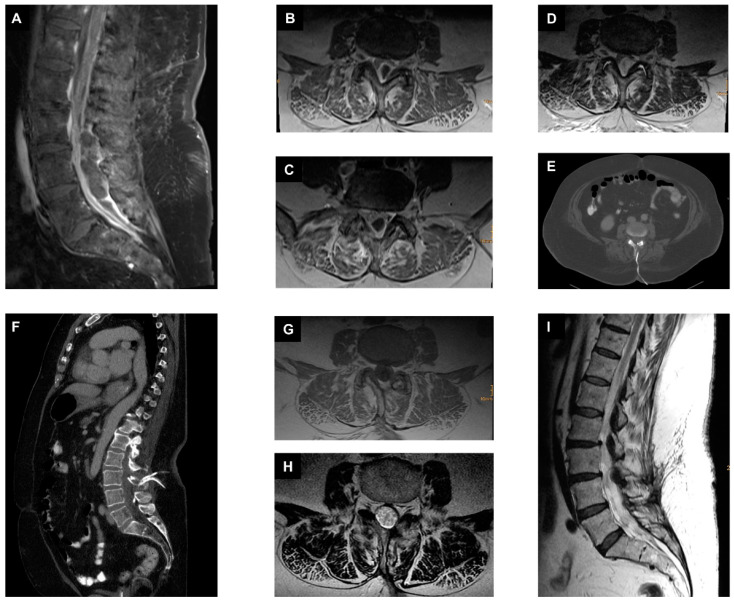
Management of ISEE with epidural suction–irrigation drainages. (**A**): A preoperative sagittal fat-saturated contrast-enhanced T1-weighted MR image of the lumbar spine shows isolated spinal epidural empyema in the dorsal part of the spinal canal at the level of L3 to S1. (**B**–**D**): Different preoperative axial T1-weighted contrast-enhanced MR images of the lumbar spine at the level of L3, L4, and L5. (**E**,**F**) Postoperative axial and sagittal reformatted CT images showing the previously placed suction–irrigation drainages cranially and caudally in the spinal canal at the level of L3/L4. (**G**): Axial T1-weighted contrast-enhanced MR image at 10 weeks at the time of antibiotic discontinuation. (**H**): Axial T2-weighted MR image at 10 weeks at the time of antibiotic discontinuation. (**I**): Sagittal T2-weighted MR image at 10 weeks at the time of antibiotic discontinuation.

Our first case was a 71-year-old female patient with distal weakness on lower extremities and urosepsis. MRI of the whole spine revealed ISEE at the level of L2 to S1. *Escherichia coli* was detected twice in blood cultures from two different peripheral regions and once in urine. Immediate microsurgical decompression with abscess evacuation and insertion of ESIDs was performed. Epidural irrigation was performed twice daily with gentamycin (5 mg in 5 mL), and samples from the irrigation drainage were collected daily before administration of local antibiotics. Once three times no bacteria were detected in the samples from the irrigation drainages, the ESIDs were removed, in this case it was the tenth postoperative day. *E. coli* was also isolated in intraoperative specimens and in the ESID samples, subsequently treated with ampicillin IV 4 g thrice daily for four weeks, and then switched to oral co-trimoxazole 160 mg/800 mg twice daily for an additional six weeks. The patient was discharged home after approximately four weeks on oral antibiotics. Follow-up showed no recurrence, so the antibiotics could be stopped after a total of ten weeks (Figure 1).

#### 2.3.2. Second Case with Spondylodiscitis and Epidural Empyema

**Figure 2:**
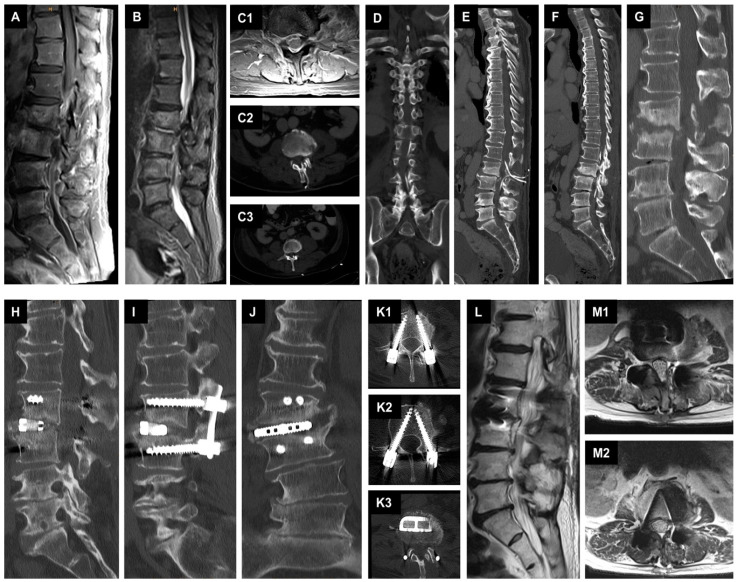
Management of SD with epidural suction–irrigation drainages. (**A**): A preoperative sagittal T1-weighted fat-saturated contrast-enhanced MR image of the lumbar spine shows spondylodiscitis at the L2/L3 level with epidural spinal empyema at the level of L2 to L5. (**B**): A preoperative sagittal T2-weighted MR image of the lumbar spine. (**C1**): Preoperative axial T1-weighted fat-saturated contrast-enhanced MR image of the level L2/L3. (**C2**–**F**): Postoperative axial, coronal, and sagittal reformatted CT images showing the placed epidural suction-irrigation drainages cranially and caudally in the spinal canal at the level of L3/L4. (**G**): Sagittal reformatted CT image showing bone destruction at the level of L2/L3 at 11 weeks at the time of proposed antibiotic discontinuation. (**H**–**K3**): Postoperative sagittal, coronal, and axial reformatted CT images showing the result of the single-stage 360-degree instrumentation in a two position. (**L**–**M2**): Sagittal and axial T2-weighted MR images displaying the result after stopping the antibiotics.

A 72-year-old male patient from a secondary hospital presented with MSSA bacteraemia detected twice in blood cultures, with increasing infection parameters, severe back pain, and immobility due to pain with lower extremities weakness that was difficult to evaluate. Therapy with intravenous flucloxacillin 2 g four times daily was started externally. Whole-spine MRI was performed showing SD at the level of L2/L3 with spinal epidural empyema at the level of L2 to L5. In addition, a calcified disc herniation at the level of L2/L3 was seen on CT with abnormal narrowing of the spinal canal. We immediately performed a microsurgical decompression at the level L2/L3 with abscess evacuation and insertion of two ESIDs. The patient was postoperatively transferred to our ICU and subsequently transferred to normal ward in stable cardiopulmonary condition. MSSA was detected twice from the ESIDs. The ESIDs were irrigated twice a day with local antibiotics. After three sterile results, the ESIDs were removed on the ninth postoperative day. After two weeks, the patient, who was in favorable general condition, had no neurologic deficits, and was able to mobilize on the ward, was transferred to a secondary hospital near to his home for continuation of antibiotic therapy. Intravenous therapy with flucloxacillin was continued for a total of six weeks and then switched to oral antibiotics for an additional five weeks. Presentation to our outpatient clinic for stopping the antibiotics with a recent MRI and CT showed increasing bone destruction at the level of L2/L3 with instability, but no epidural empyema was noted. We discontinued the antibiotics for two days. After a two-day antibiotic-free period, we performed an extreme lateral interbody fusion (XLIF) on the right side at the level L2/L3 with specimen collection followed by dorsal instrumentation in terms of a single-stage 360-degree instrumentation in a two-position. Intraoperative findings showed an abscess-free situs after SD with reactive tissue. The pathogen could not be detected in the blood cultures and in the intraoperative specimens. The patient received two weeks additional intravenous antibiotic treatment with flucloxacillin, and rifampicin followed by four weeks more of oral levofloxacin and rifampicin. After completion of antibiotics, the patient presented to our outpatient clinic with a recent MRI and CT of the lumbar spine. This showed favorable results with no evidence of relapse. The patient stated well-being and showed absence of any weakness on the lower extremities and the infection parameters were normal (Figure 2).

### 2.4. Statistical Analysis

Statistical analysis of the data was performed using the SPSS software package (SPSS Statistics 29, IBM, Armonk, NY, USA). Descriptive statistics were used, and categorical variables were tested by Fisher exact tests or chi-square tests. Numerical variables were analyzed with Mann–Whitney U tests. All statistical tests were two-sided, and a *p* value *p* < 0.05 was considered statistically significant. The dependent variable was treatment failure in SD patients, and a backward multivariate binary logistic regression analysis was performed. In this way, the regression equation was established and the relative risk value for treatment failure (odds ratio) and 95% confidence interval (CI) were determined.

## 3. Results

### 3.1. General Baseline Characteristics

Our study included 208 patients (men: 136, 65.4% vs. women: 72, 34.6%, *p* < 0.001) with PSI, of whom 142 patients presented with SD (68.3%) and 66 patients with ISEE (31.7%). In our collectives, 146 patients (70.2%) received intraoperative ESID and 62 patients (29.8%) were not treated with ESID. A total of 61.3% of SD patients were treated with ESID (87 patients), while the remaining 55 SD patients (38.7%) were treated with NON-ESID. The ISEE group was 89.4% managed with ESID (59 patients) and only 7 patients with NON-ESID. ISEE-NON-ESID group was small (7 patients), therefore only the SD patients were analyzed in detail.

### 3.2. ESID Group vs. NON-ESID Group

#### 3.2.1. Baseline Factors

The ESID and NON-ESID group did not differ in age distribution over 65 years (ESID: 74, 50.7% vs. NON-ESID: 39, 62.9%, *p* = 0.0128) and gender (ESID: 55 females, 37.7% vs. NON-ESID: 17 females 27.4%, *p* = 0.202) (Table 1). ESID group was more frequently treated with EAT strategy than NON-ESID (ESID: 105, 71.9% vs. 35, 56.5%, *p*= 0.036). Paravertebral psoas abscesses (ESID: 92, 63.0% vs. NON-ESID: 36, 58.1%, *p* = 0.535) and pleural abscesses (ESID: 28, 19.2% vs. NON-ESID: 14, 22.2%, *p* = 0.576) were found equally frequently in both groups. The distribution of detected infections in the cervical spine (CS), thoracic spine (TS), lumbar spine (LS), and in more than one part of the spine was equally common in both groups. MSSA was detected more frequently in ESID than in NON-ESID (ESID: 82/135, 60.7% vs. NON-ESID: 18/52, 34.6%, *p* = 0.002). Other bacterial groups (Streptococci/Enterococci, Enterobacterales, and coagulase-negative Staphylococci (CoNS)) did not show different distribution in both groups. Likewise, there were no differences in the distribution of the primary source of infection in the two groups (*p* = 0.143). The time from suspicion of SD or ISEE on imaging to performing surgery was significantly shorter in the ESID group (ESID: 2 [1–4] d vs. NON-ESID: 4 [1–13] d, *p* < 0.001). The time from suspicion of SD or ISEE to isolation of the pathogen was equally frequent in both groups (ESID: 4 [3–7] d vs. 5 [3–10] d, *p* = 0.198). Incidental dural tear during surgery was distributed equally in both groups (ESID: 16, 11% vs. NON-ESID: 10, 16.1%, *p*= 0.360). In the ESID group, intraoperative local antibiotic irrigation with gentamycin, vancomycin, or both was performed more frequently (ESID: 129, 88.4% vs. NON-ESID: 44, 71.0%, *p* = 0.004). Patients with ESID underwent more frequently abscess evacuation only (ESID: 82, 56.2% vs. NON-ESID: 11, 17.7%, *p* < 0.001) and more often two-stage surgery (ESID: 32, 21% vs. 5, 8.1%, *p* = 0.017). One-stage surgery was performed more frequently in patients with NON-ESIDS (ESID: 32, 21.9% vs. 46, 74.2%, *p* < 0.001).

#### 3.2.2. Risk Factors

Risk factors such as immunosuppression (ESID: 22, 15.1% vs. 13, 21.0%, *p* = 0.315), diabetes mellitus (ESID: 52, 36.6% vs. 25, 40.3%, *p* = 0.534), obesity (ESID: 48, 32.9% vs. 18, 29.0%, *p* = 0.628), malignancy (ESID: 25, 17.1% vs. 13, 21.0%, *p* = 0.558), hepatic cirrhosis (ESID: 32, 21.9% vs. 13, 21.0%, *p* = 1.000), dialysis (ESID: 5, 3.4% vs. 3, 4.8%, *p* = 0.698), presence of a stent or vascular prosthesis (ESID: 13, 8.9% vs. 4, 6.5%, *p* = 0.783), osteoporosis (ESID: 51/129, 39.5% vs. 31/60, 51.7%, *p* = 0.156), rheumatoid arthritis or increased rheumatoid factors (ESID: 29/67, 43.3% vs. 8/24, 33.3%, *p* = 0.472), gout or increased uric acid (ESID: 28/63, 44.4% vs. 17/29, 58.6%, *p* = 0.263), chronic venous insufficiency (ESID: 5, 3.4% vs. 2, 3.2%, *p* = 1.000), peripheral artery disease (ESID: 7, 4.8% vs. 6, 9.7%, *p* = 0.214), and atrial fibrillation (ESID: 31, 21.2% vs. 20, 32.3%, *p* = 0.113) were equally distributed in both groups (Table 2).

#### 3.2.3. Disease-Related Complications

SSI (ESID: 29, 19.9% vs. NON-ESID: 4, 6.5%, *p* = 0.021) and reoperations due to SSI (ESID: 24, 16.4% vs. NON-ESID: 3, 4.8%, *p* = 0.024) were significantly more common in the ESID group than in the NON-ESID group (Table 3). We found no differences in both groups in terms of complications such as sepsis (ESID: 69, 47.3% vs. NON-ESID: 37, 59.7%, *p* = 0.129), septic embolism (ESID: 34/108, 31.5% vs. NON-ESID: 22/57, 38.6%, *p* = 0.390), meningism (ESID: 22/141, 15.6% vs. NON-ESID: 15/61, 24.6%, *p* = 0.165), endocarditis with vegetation (ESID: 17/121, 14.0% vs. NON-ESID: 7/56, 12.5%, *p* = 1.000), reoperations due to persistence or instability of empyema (ESID: 41, 28.1% vs. NON-ESID: 12, 19.4%, *p* = 0.225), recurrence rate (ESID: 25/98, 25.5% vs. NON-ESID: 6/40, 15.0%, *p* = 0.261), mortality (ESID: 6, 4.1% vs. NON-ESID: 6, 9.7%, *p* = 0.189), hospital stay (ESID: 31 [22–48] d vs. NON-ESID: 35 [24–490] d, *p* = 0.512), ICU stay (ESID: 1 [0–8] d vs. 2 [0–13] d, *p* = 0.423), intravenous antibiotic duration (ESID: 4 [3–6] w vs. NON-ESID: 4 [3–6] w, *p* = 0.426), and total antibiotic duration (ESID: 8 [6–12] w vs. NON-ESID: 10 [6–12] w, *p* = 0.301).

### 3.3. ESID Group vs. NON-ESID Group in Spondylodiscitis

#### 3.3.1. Baseline Factors

Age over 65 years, gender, EAT/TAT, paravertebral psoas abscesses, pleural abscesses, and distribution in the spine (CS, TS, LS, and more than part of the spine) were equally distributed in the ESID and NON-ESID groups (Table 4). MSSA were detected more frequently in patients with ESID than in patients without ESID (ESID: 44/80, 55.0% vs. NON-ESID: 13/45, 28.9%, *p* = 0.005); however, Streptococci/Enterococci, Enterobacterales, and CoNS showed no differences between the two groups. The primary sources of infection were equally distributed in both groups (*p* = 0.0676). The time to surgery was significantly longer in the NON-ESID group than in the ESID group (ESID: 2 [1–6] d vs. NON-ESID: 5 [2–14] d, *p* = 0.004), although the time to pathogen detection was the same in both groups (ESID: 5 [3–8] d vs. NON-ESID: 6 [3–11] d, *p* = 0.470). There were no differences between both groups in incidental dural tears (ESID: 11, 12.6% vs. NON-ESID: 8, 14.5%, *p* = 0.803). Patients with ESID were more likely to be irrigated intraoperatively with a local antibiotic (ESID: 80, 92.0% vs. NON-ESID: 40, 72.7%, *p* = 0.004). ESID patients underwent more often abscess evacuation only (ESID: 25, 28.7% vs. NON-ESID: 5, 9.1%, *p* < 0.006) and more often two-stage surgery (ESID: 32, 36.8% vs. 5, 9.1%, *p* < 0.001). One-stage surgery was performed more frequently in patients with NON-ESIDS (ESID: 30, 34.5% vs. 45, 81.8%, *p* < 0.001).

#### 3.3.2. Risk Factors

Patients with ESID were less frequently to have artificial valve replacement than patients without ESID (ESID: 3, 3.4% vs. 7, 12.7%, *p* = 0.046). No differences were seen between the two groups for other risk factors (Table 5).

#### 3.3.3. Disease-Related Complications

SSI (ESID: 22, 25.3% vs. NON-ESID: 3, 5.5%, *p* = 0.003), reoperations due to SSI (ESID: 20, 23.0% vs. NON-ESID: 2, 3.6%, *p* = 0. 002), and reoperations due to empyema persistence or instability (ESID: 37, 42.5% vs. NON-ESID: 12, 21.8%, *p* = 0.012) were significantly more frequent in the ESID group than in the NON-ESID group (Table 6). The relapse rate was also higher in patients with ESID than in patients with NON-ESID (ESID: 21, 37.5% vs. NON-ESID: 6, 16.7%, *p* = 0.037). No differences were found between the two groups for other disease-related complications.

### 3.4. Success Rate

**Figure 3:**
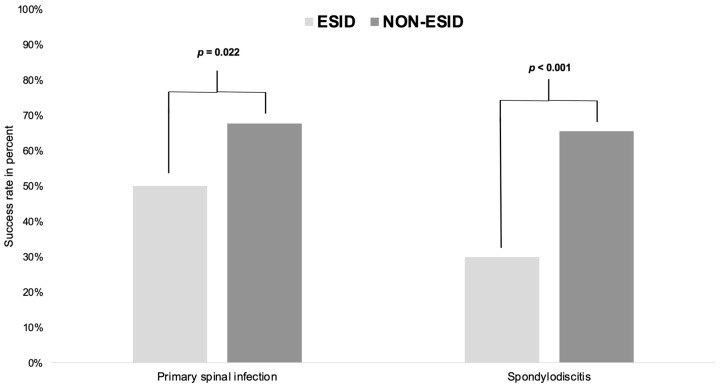
Success rate in patients with primary spinal infection or spondylodiscitis treated with/without ESID. ESID: epidural suction–irrigation drainage group; NON-ESID: group without epidural suction–irrigation drainage; The *p*-values are significant when *p* < 0.05, as indicated in the methods according to the Fisher exact test.

Success rate was defined as a single (one- or two-stage surgical strategy) uncomplicated ESID or NON-ESID treatment. Treatment failure was present in persistent empyema, instability, or SSI with or without reoperation, and relapse or fatality. Overall, the success rate was lower in PSI patients with ESID than NON-ESID patients (ESID: 73, 50.0% vs. NON-ESID: 42, 67.7%, *p* = 0.022). SD patients without ESID showed a better success rate than patients with ESID (ESID: 26, 29.9% vs. NON-ESID: 36, 65.6%, *p* < 0.001) (Table 7, Figure 3).

### 3.5. Multivariate Analyses

Multivariate binary logistic regression analyses are summarized in Table 8. Treatment with ESID (*p* < 0.001; OR: 0.201; 95% CI: 0.089–0.451) was identified as a significant independent risk factor for treatment failure in patients with SD in our cohort.

Sex (*p* = 0.113; OR: 0.487; 95% CI: 0.200–1.185), age over 65 years (*p* = 0.311; OR: 0.629; 95% CI: 0.257–1.542), MSSA (*p* = 0.311; OR: 0.928; 95% CI: 0.370–2.329), time to pathogen detection (*p* = 0.058; OR: 0.938; 95% CI: 0.877–1.002), EAT (*p* = 0.211; OR: 1.707; 95% CI: 0.738–3.948), time to surgery (*p* = 0.748; OR: 0.992; 95% CI: 0.947–1.040), diabetes mellitus (*p* = 0.251; OR: 0.612; 95% CI: 0.265–1.415), hepatic cirrhosis (*p* = 0.837; OR: 0.895; 95% CI: 0.312–2.568), malignancy (*p* = 0.286; OR: 0.539; 95% CI: 0.174–1.676), paravertebral psoas abscess (*p* = 0.067; OR: 0.468; 95% CI: 0.208–1.053), pleural abscess (*p* = 0.141; OR: 0.472; 95% CI: 0.174–1.281), and incidental dural tears (*p* = 0.127; OR: 0.382; 95% CI: 0.111–1.315) showed no significance in multivariate analysis.

## 4. Discussion

The key message of this study and our 20-year experience with the ESID technique showed that ESID did not confer any benefits in SD patients, possibly even disadvantages in terms of worsening short- and long-term outcomes, such as required reoperations and increased relapse rate. In the ISEE patients, we were unable to perform any meaningful comparisons between ESID and NON-ESID patients due to the small number of ISEE patients. The adverse effects in SD patients were mainly due to SSI and local persistence of infection, probably caused by seroma formation.

***Baseline factors:***

The following baseline factors were equally distributed between both ESID and NON-ESID, even when considering SD patients separately: Age over 65 years, gender, paravertebral psoas abscess or pleural abscess, location in the spine, bacterial groups such as Streptococci and Enterococci, Enterobacterales, CoNS, primary sources of infection, time to pathogen detection, and incidental dural tears.

In total, ESID patients were more often treated with EAT than TAT compared to NON-ESID. In SD patients alone, we found no difference between the ESID and NON-ESID in terms of TAT/EAT. Overall, the number of patients with EAT in our cohort is greater than with TAT because patients were referred to us for immediate surgical treatment and had EAT upfront.

Baseline factors such as MSSA bacterial group, time to surgery, intraoperative antibiotic irrigation, and type of surgery (abscess evacuation only, one- or two-stage surgery) showed a difference between ESID and NON-ESID in PSI and SD patients alone.

In SD patients, MSSA were detected more frequently in the ESID group than in the NON-ESID group, although this may be an incidental finding since MSSA is a typical pathogen of SD [12,13,14].

In our SD cohort, the time from the suspected clinical radiological diagnosis to surgery was shorter in the ESID group than in the NON-ESID group. Authors reported that early surgery in SD showed better clinical outcomes [15]. Thus, the ESID group in SD has an advantage over NON-ESID, if the assumption were accurate. The time from symptom onset to first surgical treatment was reported differently in the literature, varying from 17 to 69 days [16,17].

More than 70% of the ESID and NON-ESID groups in SD patients were irrigated intraoperatively with local antibiotics, which may have affected the outcomes; however, the ESID group was irrigated intraoperatively more frequently than the NON-ESID group.

The NON-ESID group was treated mainly with single-stage surgery, whereas the three procedures were equally distributed in the ESID group. In a retrospective study of 118 patients, Bydon et al. reported no differences in recurrence and revision rates in patients with abscess evacuation and instrumentation and patients with only abscess evacuation [18]. Dietz et al. demonstrated significant benefits of additive fusion in a population of 2662 patients in terms of recurrence, revision rate, and postoperative complications [19].

***Risk factors:***

Risk factors such as immunosuppression, diabetes mellitus, obesity, malignancy, liver cirrhosis, dialysis, stent or vascular prosthesis, osteoporosis, rheumatoid arthritis or increased rheumatoid factors, gout or increased uric acid, chronic venous insufficiency, peripheral arterial disease, and atrial fibrillation showed no between-group differences. Artificial heart valve replacement was found more frequently in SD patients in the NON-ESID group than in the ESID group.

***Disease-related complications:***

We found a significant increased SSI (19.9%) with required revisions due to SSI (16.4%) in ESID patients with PSI compared to NON-ESID patients with PSI (SSI: 6.5%, revision due to SSI: 4.8%). The ESID technique carries an increased risk of epidural fluid stasis due to irrigation [11], which probably led to SSI.

However, all other disease-related complications such as sepsis, septic embolism, meningism, endocarditis with vegetations, reoperation for persistent empyema or increased spinal instability, relapse rate, mortality, length of hospital and ICU stay, duration of intravenous antibiotic administration, and total duration of antibiotic administration were equally distributed between ESID and NON-ESID patients with PSI in our cohort.

SD cohort showed an increased rate of SSI, relapse, revisions due to SSI, persistent empyema, or instability in ESID patients compared with NON-ESID.

***Success rate:***

ESID showed a worse success rate in PSI and especially in SD patients, and the inefficacy of this technique can only be explained by the accumulation of fluid with the formation of seromas and the resulting increasing bacterial load.

***Multivariate analyses:***

The multivariate regression analysis showed that ESID technique was an independent risk factor for treatment failure in patients with SD, which was suggested in the previous study by Löhr [11].

***Limitations and strengths of this study***

Our study is limited by its retrospective design and a possible selection bias toward more severe cases due to the high degree of specialization at our university center. The number of ISEE patients treated with NON-ESID is very limited, with only seven patients. Nevertheless, our data are derived from detailed clinical, imaging, and microbiological state-of-the-art diagnostic assessment with high internal validity and a meaningful sample size. Finally, the generalizability of our observations is limited by the monocentric design of our study.

## 5. Conclusions

Our retrospective cohort study and more than 20 years of experience with the ESID technique suggest a negative effect in patients with SD in terms of SSI and relapse rate. The evaluation of ISEE patients is limited due to the small number of NON-ESID patients. In SD patients, the unfavorable effects were mainly due to SSI and local persistence of infection, probably caused by seroma formation.

## Figures and Tables

**Table 1:** Baseline factors between ESID and NON-ESID.

Baseline Factors	ESID (n = 146, 70.2%)	NON-ESID (n = 62, 29.8%)	*p*-Value
Age > 65 years	74/146 (50.7%)	39/62 (62.9%)	0.128 ^(1)^
Gender	F: 55 vs. M: 91	F: 17 vs. M: 45	0.202 ^(1)^
EAT	105/146 (71.9%)	35/62 (56.5%)	**0.036 ^(1)^**
TAT	41/146 (28.1%)	27/62 (43.5%)	
Paravertebral psoas abscesses	92/146 (63.0%)	36/62 (58.1%)	0.535 ^(1)^
Pleural abscesses	28/146 (19.2%)	14/62 (22.6%)	0.576 ^(1)^
CS	43/146 (29.5%)	18/62 (29.0%)	1.000 ^(1)^
TS	58/146 (39.7%)	24/62 (38.7%)	1.000 ^(1)^
LS	101/146 (69.2%)	36/62 (58.1%)	0.150 ^(1)^
More than one part of spine	43/146 (29.5%)	14/62 (22.6%)	0.396 ^(1)^
MSSA (n = 187)	82/135 (60.7%)	18/52 (34.6%)	**0.002 ^(1)^**
SE (n = 187)	19/135 (14.1%)	12/52 (23.1%)	0.187 ^(1)^
Enterobacterales (n = 187)	12/135 (8.9%)	8/52 (15.4%)	0.198 ^(1)^
CoNS (n = 187)	12/135 (8.9%)	8/52 (15.4%)	0.198 ^(1)^
Primary infectious sources (n = 145)	--	--	0.143 ^(2)^
Time to surgery	2 [1–4] d *	4 [1–13] d *	**<0.001 ^(2)^**
Time to pathogen detection	4 [3–7] d *	5 [3–10] d *	0.198 ^(2)^
Incidental dural tears	16/146 (11.0%)	10/62 (16.1%)	0.360 ^(1)^
Intraoperative antibiotic Irrigation	129/146 (88.4%)	44/62 (71.0%)	**0.004 ^(1)^**
Only abscess evacuation	82/146 (56.2%)	11/62 (17.7%)	**<0.001 ^(1)^**
One-stage surgery	32/146 (21.9%)	46/62 (74.2%)	**<0.001 ^(1)^**
Two-stage surgery	32/146 (21.9%)	5/62 (8.1%)	**0.017 ^(1)^**

ESID: epidural suction–irrigation drainage group; NON-ESID: group without epidural suction–irrigation drainage; EAT: empirical antibiotic treatment; TAT: targeted antibiotic treatment; CS: cervical spine, TS: thoracic spine; LS: lumbar spine; MSSA: methicillin-sensitive *Staphylococcus* aureus; SE: *Streptococcus* spp. and *Enterococcus* spp.; CoNS: coagulase-negative *Staphylococci*; *: median [interquartile range]; (1): Fisher exact test; (2): Mann–Whitney U test. One-stage surgery: simultaneous abscess evacuation and fixation; Two-stage surgery: initial abscess evacuation and then fixation. Bold values are significant results (*p* < 0.05) as indicated in the methods.

**Table 2:** Risk factors between ESID and NON-ESID.

Risk Factors	ESID (n = 146, 70.2%)	NON-ESID (n = 62, 29.8%)	*p*-Value
Immunosuppression	22/146 (15.1%)	13/62 (21.0%)	0.315 ^(1)^
Diabetes mellitus	52/146 (36.6%)	25/62 (40.3%)	0.534 ^(1)^
Obesity (BMI > 30 kg/m^2^)	48/146 (32.9%)	18/62 (29.0%)	0.628 ^(1)^
Malignancy	25/146 (17.1%)	13/62 (21.0%)	0.558 ^(1)^
Hepatic cirrhosis	32/146 (21.9%)	13/62 (21.0%)	1.000 ^(1)^
Dialysis	5/146 (3.4%)	3/62 (4.8%)	0.698 ^(1)^
Stent or vascular prosthesis	13/146 (8.9%)	4/62 (6.5%)	0.783 ^(1)^
Artificial heart valve replacement	5/146 (3.4%)	7/62 (11.3%)	**0.045 ^(1)^**
Osteoporosis (n = 189)	51/129 (39.5%)	31/60 (51.7%)	0.156 ^(1)^
RA or increased RF (n = 91)	29/67 (43.3%)	8/24 (33.3%)	0.472 ^(1)^
Gout or increased uric acid (n = 92)	28/63 (44.4%)	17/29 (58.6%)	0.263 ^(1)^
Chronic venous insufficiency	5/146 (3.4%)	2/62 (3.2%)	1.000 ^(1)^
Peripheral artery disease	7/146 (4.8%)	6/62 (9.7%)	0.214 ^(1)^
Atrial fibrillation	31/146 (21.2%)	20/62 (32.3%)	0.113 ^(1)^

ESID: epidural suction–irrigation drainage group; NON-ESID: group without epidural suction–irrigation drainage; BMI: body mass index; RA: rheumatoid arthritis; RF: rheumatoid factors; ^(1)^: Fisher exact test. Bold values are significant results (*p* < 0.05) as indicated in the methods.

**Table 3:** Disease-related complications between ESID and NON-ESID.

Variable	ESID (n = 146, 70.2%)	NON-ESID (n = 62, 29.8%)	*p*-Value
Sepsis	69/146 (47.3%)	37/62 (59.7%)	0.129 ^(1)^
Septic embolism (n = 165)	34/108 (31.5%)	22/57 (38.6%)	0.390 ^(1)^
Meningism (n = 202)	22/141 (15.6%)	15/61 (24.6%)	0.165 ^(1)^
Endocarditis with vegetation (n = 177)	17/121 (14.0%)	7/56 (12.5%)	1.000 ^(1)^
SSI	29/146 (19.9%)	4/62 (6.5%)	**0.021 ^(1)^**
Reoperation due SSI	24/146 (16.4%)	3/62 (4.8%)	**0.024 ^(1)^**
Reoperation (empyema or instability)	41/146 (28.1%)	12/62 (19.4%)	0.225 ^(1)^
Relapse rate (n = 138)	25/98 (25.5%)	6/40 (15.0%)	0.261 ^(1)^
Mortality	6/146 (4.1%)	6/62 (9.7%)	0.189 ^(1)^
Hospital stay	31 [22–48] d *	35 [24–49] d *	0.512 ^(2)^
ICU stay	1 [0–8] d *	2 [0–13] d *	0.423 ^(2)^
Intravenous antibiotic duration	4 [3–6] w *	4 [3–6] w *	0.426 ^(2)^
Total antibiotic duration	8 [6–12] w *	10 [6–12] w *	0.301 ^(2)^

ESID: epidural suction–irrigation drainage group; NON-ESID: group without epidural suction–irrigation drainage; SSI: surgical site infection; ICU: intensive care unit; *: median [interquartile range]; (1): Fisher exact test; (2): Mann–Whitney U test. Bold values are significant results (*p* < 0.05) as indicated in the methods.

**Table 4:** Baseline factors between ESID and NON-ESID in SD.

Baseline Factors	SD (n = 142, 68.3%)		
	ESID (n = 87, 61.3%)	NON-ESID (n = 55, 38.7%)	*p*-Value
Age > 65 years	53/87 (60.9%)	35/55 (63.6%)	0.859 ^(1)^
Gender	F: 29 vs. M: 58	F: 12 vs. M: 43	0.184 ^(1)^
EAT	58/87 (66.7%)	31/55 (56.4%)	0.285 ^(1)^
TAT	29/87 (33.3%)	24/55 (43.6%)	
Paravertebral psoas abscesses	62/87 (71.3%)	33/55 (60.0%)	0.201 ^(1)^
Pleural abscesses	20/87 (23.0%)	13/55 (23.6%)	1.000 ^(1)^
CS	24/87 (27.6%)	15/55 (27.3%)	1.000 ^(1)^
TS	30/87 (34.5%)	19/55 (34.5%)	1.000 ^(1)^
LS	62/87 (71.3%)	34/55 (61.8%)	0.272 ^(1)^
More than one part of spine	25/87 (28.7%)	11/55 (20.0%)	0.322 ^(1)^
MSSA (n= 187)	44/80 (55.0%)	13/45 (28.9%)	**0.005 ^(1)^**
SE (n = 187)	14/80 (17.5%)	12/45 (26.7%)	0.255 ^(1)^
Enterobacterales (n= 187)	8/80 (10.0%)	8/45 (17.8%)	0.267 ^(1)^
CoNS (n = 187)	8/80 (10.0%)	7/45 (15.6%)	0.397 ^(1)^
Primary infectious sources (n = 145)	--	--	0.676 ^(2)^
Time to surgery	2 [1–6] d *	5 [2–14] d *	**0.004 ^(2)^**
Time to pathogen detection	5 [3–8] d *	6 [3–11] d *	0.470 ^(2)^
Incidental dural tears	11/87 (12.6%)	8/55 (14.5%)	0.803 ^(1)^
Intraoperative antibiotic Irrigation	80/87 (92.0%)	40/55 (72.7%)	**0.004 ^(1)^**
Only abscess evacuation	25/87 (28.7%)	5/55 (9.1%)	**0.006 ^(1)^**
One-stage surgery	30/87 (34.5%)	45/55 (81.8%)	**<0.001 ^(1)^**
Two-stage surgery	32/87 (36.8%)	5/55 (9.1%)	**<0.001 ^(1)^**

ESID: epidural suction–irrigation drainage group; NON-ESID: group without epidural suction–irrigation drainage; SD: spondylodiscitis; EAT: empirical antibiotic treatment; TAT: targeted antibiotic treatment; CS: cervical spine, TS: thoracic spine; LS: lumbar spine; MSSA: methicillin-sensitive *Staphylococcus* aureus; SE: *Streptococcus* spp. and *Enterococcus* spp.; CoNS: coagulase-negative *Staphylococci*; *: median [interquartile range]; One-stage surgery: simultaneous abscess evacuation and fixation; Two-stage surgery: initial abscess evacuation and then fixation. (1): Fisher exact test; (2): Mann–Whitney U test. Bold values are significant results (*p* < 0.05) as indicated in the methods.

**Table 5:** Risk factors between ESID and NON-ESID in SD.

Risk Factors	SD (n = 142, 68.3%)		
	ESID (n = 87, 61.3%)	NON-ESID (n = 55, 38.7%)	*p*-Value
Immunosuppression	16/87 (18.4%)	12/55 (21.8%)	0.668 ^(1)^
Diabetes Mellitus	35/87 (40.2%)	24/55 (43.6%)	0.729 ^(1)^
Obesity (BMI > 30 kg/m^2^)	23/87 (26.4%)	14/55 (25.5%)	1.000 ^(1)^
Malignancy	14/87 (16.1%)	12/55 (21.8%)	0.505 ^(1)^
Hepatic cirrhosis	25/87 (28.7%)	13/55 (23.6%)	0.563 ^(1)^
Dialysis	4/87 (4.6%)	3/55 (5.5%)	1.000 ^(1)^
Stent or vascular prosthesis	11/87 (12.6%)	4/55 (7.3%)	0.406 ^(1)^
Artificial heart valve replacement	3/87 (3.4%)	7/55 (12.7%)	**0.046 ^(1)^**
Osteoporosis (n = 189)	33/76 (43.4%)	30/55 (54.5%)	0.221 ^(1)^
RA or increased RF (n = 91)	20/44 (45.5%)	8/22 (36.4%)	0.600 ^(1)^
Gout or increased uric acid (n = 92)	19/42 (45.2%)	17/29 (58.6%)	0.337 ^(1)^
Chronic venous insufficiency	1/87 (1.1%)	2/55 (3.6%)	0.560 ^(1)^
Peripheral artery disease	5/87 (5.7%)	6/55 (10.9%)	0.337 ^(1)^
Atrial fibrillation	23/87 (26.4%)	20/55 (36.4%)	0.261 ^(1)^

ESID: epidural suction–irrigation drainage group; NON-ESID: group without epidural suction–irrigation drainage; SD: spondylodiscitis; BMI: body mass index; RA: rheumatoid arthritis; RF: rheumatoid factors; (1): Fisher exact test. Bold values are significant results (*p* < 0.05) as indicated in the methods.

**Table 6:** Disease-related complications between ESID and NON-ESID in SD.

Variable	SD (n = 142, 68.3%)		
	ESID (n = 87, 61.3%)	NON-ESID (n = 55, 38.7%)	*p*-Value
Sepsis	59/87 (67.8%)	35/55 (37.2%)	0.716 ^(1)^
Septic embolism (n = 165)	31/67 (46.3%)	21/50 (42.0%)	0.709 ^(1)^
Meningism (n = 202)	12/83 (14.5%)	13/54 (24.1%)	0.178 ^(1)^
Endocarditis with vegetation (n = 177)	16/75 (21.3%)	7/50 (14.0%)	0.352 ^(1)^
SSI	22/87 (25.3%)	3/55 (5.5%)	**0.003 ^(1)^**
Reoperation due SSI	20/87 (23.0%)	2/55 (3.6%)	**0.002 ^(1)^**
Reoperation (empyema or instability)	37/87 (42.5%)	12/55 (21.8%)	**0.012 ^(1)^**
Relapse rate (n = 138)	21/56 (37.5%)	6/36 (16.7%)	**0.037 ^(1)^**
Mortality	5/87 (5.7%)	6/55 (10.9%)	0.337 ^(1)^
Hospital stay	40 [31–56] d *	35 [26–51] d *	0.139 ^(2)^
ICU stay	3 [0–13] d *	2 [0–12] d *	0.893 ^(2)^
Intravenous antibiotic duration	5 [4–6] w *	4 [3–7] w *	0.189 ^(2)^
Total antibiotic duration	10 [8–12] w *	10 [7–12] w *	0.789 ^(2)^

ESID: epidural suction–irrigation drainage group; NON-ESID: group without epidural suction–irrigation drainage; SD: spondylodiscitis; SSI: surgical site infection; ICU: intensive care unit; *: median [interquartile range]; (1): Fisher exact test; (2): Mann–Whitney U test. Bold values are significant results (*p* < 0.05) as indicated in the methods.

**Table 7:** Success rate in patients with PSI or SD treated with/without ESID.

Infection	ESID	NON-ESID	*p*-Value
Primary spinal infection (n = 208)	73/146 (50.0%)	42/62 (67.7%)	**0.022 ^(1)^**
Spondylodiscitis (n = 142)	26/87 (29.9%)	36/55 (65.5%)	**<0.001 ^(1)^**

ESID: epidural suction–irrigation drainage group; NON-ESID: group without epidural suction–irrigation drainage; (1): Fisher exact test. Bold values are significant results (*p* < 0.05) as indicated in the methods.

**Table 8:** Multivariate binary logistic regression analysis to identify independent risk factors for treatment failure in patients with SD.

Variables	Multivariate Logistic Regression	
	OR (95% CI)	*p*-Value
Males	0.487 (0.200–1.185)	0.113
Age > 65 years	0.629 (0.257–1.542)	0.311
Methicillin-sensitive *Staphylococcus* aureus	0.928 (0.370–2.329)	0.874
Time to pathogen detection	0.938 (0.877–1.002)	0.058
Empirical antibiotic therapy	1.707 (0.738–3.948)	0.211
Time to surgery	0.992 (0.947–1.040)	0.748
Diabetes mellitus	0.612 (0.265–1.415)	0.251
Hepatic cirrhosis	0.895 (0.312–2.568)	0.837
Malignancy	0.539 (0.174–1.676)	0.286
Paravertebral psoas abscess	0.468 (0.208–1.053)	0.067
Pleural abscess	0.472 (0.174–1.281)	0.141
Incidental dural tears	0.382 (0.111–1.315)	0.127
Epidural suction-irrigation drainage	0.201 (0.089–0.451)	**<0.001**

OR: odds ratio, CI: confidence interval. Bold values are significant results (*p* < 0.05) as indicated in the methods.

## Data Availability

The original contributions presented in the study are included in the article; further inquiries can be directed to the corresponding author/s.
